# The Evolving Concept of Poor-Prognosis for Women Undertaking IVF and the Notion of Growth Hormone as an Adjuvant; A Single-Center Viewpoint

**DOI:** 10.3389/fendo.2019.00808

**Published:** 2019-11-20

**Authors:** John L. Yovich, Yun Ye, Sheena L. P. Regan, Kevin Noel Keane

**Affiliations:** ^1^PIVET Medical Centre, Perth, WA, Australia; ^2^Department of Pharmacy and Biomedical Sciences, Faculty of Health Sciences, Curtin University, Perth, WA, Australia; ^3^Zhongshan People's Hospital, Zhongshan, China

**Keywords:** poor-prognosis, IVF adjuvants, poor ovarian responder (POR), growth hormone (GH), adult growth hormone deficiency (AGHD), oocyte utilization rate, embryo utilization rate, live birth productivity rate

## Abstract

IVF is currently regarded as a successful new technology with the number of IVF children currently well over 8 million worldwide. This has been achieved by an explosive plethora of facilities. However, from its earliest history, IVF has been beset by poor-prognosis on a treatment cycle basis, an aspect which has been a constant feature for the majority of treatments to this stage. The 2019 Australian and New Zealand Assisted Reproduction Database (ANZARD) report shows that IVF clinics have live birth productivity rates (from combined initiated fresh and frozen cycles) ranging from 9.3 to 33.2%. Over the past 40 years there have been a number of innovations which have steadily moved the success rates forward, but progress is held back by an intransigent group of women who can be classified as being poor-prognosis from one or more adverse factors, namely advanced age (>40 years), poor ovarian response (POR) to ovarian stimulation, inability to generate high quality blastocyst-stage embryos, recurrent implantation failure, or recurrent early pregnancy losses. A number of strategies are variously applied including the use of recombinant growth hormone (GH) adjuvant therapy. Our retrospective studies at PIVET over the past decade show a 6.2-fold chance of live birth for fresh cycle embryo transfers following GH injections of 1–1.5 IU daily given for 3–6 weeks in the lead-up to the trigger for ovum pick-up. We have also recently reported the live birth rates from frozen embryo transfers utilizing those blastocyst embryos generated under GH influence and showed the live birth rate was 2.7-fold higher in a carefully matched poor-prognosis group. This experience has been compared to the total 42 GH studies reported since the year 2000, the majority matching those of PIVET with significant increases in both oocyte and embryo utilization rates but only ~50% are followed by elevated live birth rates. We argue that this discrepancy relates to failure in addressing other causes of poor-prognosis along with the wastage of transferring more than a single embryo in the fresh cycle, when ANZARD data indicates a significantly higher chance of live birth from frozen embryo transfers.

## Introduction

The concept of poor-prognosis for women undertaking *in-vitro* fertilization (IVF) is embedded in the early history of human IVF and continues to be a stubborn but evolving concept. One attempt to define the poor-prognosis woman focussed on those with low ovarian reserve who therefore had limited response to ovarian stimulation strategies, even applying maximal dosage of gonadotrophins. The ESHRE working group (1) categorized a poor ovarian responder (POR), applying a definition where women had at least two of the following three features:

- Advanced maternal age (≥40 years) or any other risk factor for POR;
- A previous POR ( ≤ 3 oocytes with a conventional stimulation protocol);
- An abnormal ovarian reserve test (ORT) i.e., antral follicle count (AFC), <5–7 follicles or anti-Mullerian hormone (AMH) level <0.5–1.1 ng/ml (For SI units; <3.5–8 pmol/L).

Added point: The ESHRE working group accepted that two episodes of POR after maximal stimulation are sufficient to define a patient as poor responder in the absence of advanced maternal age or abnormal ORT.

“By definition, the term POR refers to the ovarian response and, therefore, one stimulated cycle is considered essential for the diagnosis of POR. However, patients over 40 years of age with an abnormal ORT may be classified as poor responders since both advanced age and an abnormal ORT may indicate reduced ovarian reserve and act as a surrogate of ovarian stimulation cycle. In this case, the patients should be more properly defined as expected PORs.”

## Poor-Prognosis vs. POR

The aforementioned Bologna definition has been criticized from the outset, mainly because it fails to address the question of oocyte quality and the relevance of risk factors which, together influence embryo quality. Furthermore, standardization of both AFC and AMH assays remains problematic (2). The Israeli group of Younis and colleagues indicated 6 main areas of debate but our view would contend that POR is but one factor under a broader problem of poor-prognosis factors limiting the chance of generating live births from IVF.

To understand poor-prognosis requires a review of the historical evolution of IVF to its current improved, but still rather imperfect, position. Furthermore, when attempting to evaluate adjuvant therapies given to women who had experienced repeated failures in IVF programmes, we faced several problems related to modern IVF evolutionary factors, namely the increased reliance on cryopreserved embryos, the progress toward single embryo transfers (preferentially undertaken at the blastocyst stage) and the methodology of evaluating embryo quality. Along with those evolutionary trends, “advanced ovarian stimulation protocols” have also emerged. For comparative evaluations of these trends new definitions have been introduced such as a Productivity Rate (3), meaning the total number of live births arising from a single IVF cycle initiated. The Productivity Rate may be classified according to a particular clinical regimen (±adjuvant therapy), or a modified laboratory protocol. Ideally, the Productivity Rate reflects the real outcome, but clinical studies are often frustrated because of an increasing trend to cryopreserve all embryos (so called “freeze-all” protocol) and embryos may remain in cryopreservation for several years, unable to be evaluated during a particular study period. Other frustrations for research studies include the 10-month long period from IVF cycle initiation (e.g., Day of commencing ovarian cycle tracking or stimulation) to birth outcome. Surrogate measures over a shorter period may prove to be valid, such as the oocyte utilization number/or rate (being the number/or proportion of oocytes which result in embryos which prove suitable for fresh-cycle transfer or cryopreservation, ideally at blastocyst-stage). Oocyte Utilization can be rated per total oocytes recovered at oocyte pick-up (OPU) or per number of 2PN-stage oocytes resulting after fertilization (such oocytes reflecting a “mature” group). This latter category may reasonably be termed Embryo Utilization Rate (3). These terms were introduced during PIVET's earliest GH-adjuvant studies (4) and have proven useful, essentially validated, in subsequent studies and reports (5, 6). Some reports use the term cumulative live birth rate (CLBR) but that term historically related to several OPU cycles, hence a specific term such as productivity rate should be preferable (3). Terminology aside, the concept is now incorporated into the annual Australian and New Zealand Assisted Reproduction Database (ANZARD) report (7) which also reveals that “freeze-all” was conducted in 24.2% of the 47,545 autologous fresh IVF cycles undertaken in 2017.

At PIVET the term poor-prognosis has been applied according to any one of 5 criteria, namely:

- All women aged 40 years and above
- All women categorized as poor-prognosis from previous IVF, meaning repeated failures (≥3) RIF
- All PORs (generating ≤4 oocytes despite FSH dosing maximized at 450 IU daily)
- All cases with “E” categorization according to PIVET FSH-dosing algorithms [AMH <5 pmol/l & AFC <5 follicles; (5)] matching ORT according to Bologna criteria
- All cases where resultant embryo quality rated poor, meaning no suitable blastocysts for cryopreservation (Good prognosis in IVF generates 8–12 oocytes resulting in ≥3 blastocysts with gradings 3BB or better).

For the purpose of this article, it can be seen that historically the diagnosis of poor-prognosis is defined after one or more IVF attempts have already been undertaken, an expensive and unhappy scenario for those patients who have failed to achieve a pregnancy and ensuing live birth. Ideally, the diagnosis should be established following primary assessment of the infertile couple, so that remedial strategies can be introduced from the outset. One of those strategies can be the application of adjuvants such as growth hormone for which the notion of adult growth hormone deficiency (AGHD) has been proposed (8).

### Historical Perspective

From the very beginning of human IVF, poor-prognosis has been intrinsic to the treatment mode. This concept of poor-prognosis has continued to change with the evolution of methodologies and technologies in IVF. The 40th birthday of the world's first IVF live birth, Louise Brown in July, 2018 was lauded world-wide as she is now accompanied by more than 8 million IVF offspring. However, it is relevant for this discussion concerning poor-prognosis in IVF, to note that her birth followed a decade of effort by the acknowledged “Fathers of IVF” whereby 282 couples underwent 457 cycles of treatment and 112 women completed an embryo transfer procedure (9). Gynecologist Patrick Steptoe, working in Oldham near Manchester UK, undertook the vast majority of oocyte pick-ups (OPUs) by laparoscopy, a novel procedure he introduced to Britain in 1967 after his training in France with Raoul Palmer, the pioneer of modern laparoscopy. There were some women who required laparotomy due to dense pelvic adhesions precluding safe access to the pelvis via laparoscopy. Physiologist Robert Edwards, from Cambridge developed the IVF protocols and performed the embryology procedures with technical assistance from nurse Jean Purdy, who later qualified as an embryologist. After many years of frustration, including several biochemical pregnancies and an ectopic pregnancy in 1975, Steptoe advised removal of, or preliminary clipping of the fallopian tubes. Around the same time, Edwards encouraged the switch from ovarian stimulation, which had attendant problems with the luteal phase, to tracking of the natural cycle. This was facilitated by introducing the very sensitive HiGonavis pregnancy kit from Mochida Pharmaceuticals which could detect both hCG and LH levels as low as 20 IU/L in urine. The final phase of their work was performed in nearby Dr. Kershaw's Cottage hospital where 79 couples were admitted and 68 women reached the stage of laparoscopy with 55 achieving successful OPU. However, only 32 cases had a Day-2 embryo suitable for transfer; of these, 4 clinical pregnancies ensued—one miscarried at 11 weeks, a second delivered pre-term at 21 weeks with neonatal demise soon after the birth. This case was categorized as a post-amniocentesis loss and caused the “Fathers” to advise against routine testing in the future. The 3rd pregnancy resulted in the delivery of Louise Brown on 25 July 1978 and the 4th resulted in the delivery of Alistair MacDonald on 14 January 1979 (9).

This historical detail is relevant for this article as 3 livebirths from 457 cycles initiated (<1%) or from 112 embryo transfers (3%) can be considered poor-prognosis by current standards. The Kershaw history was somewhat better as the pregnancy rate was 5% of the 79 women admitted (initiated), 6% of the 68 laparoscopies, 7% of OPUs, and 12.5% of ETs. Progressing beyond the perinatal phase, and depending on definition for livebirth dating being either 20 or 28 weeks, the live birth rate was 2 for the 79 women initiated (2.5%). These results have stimulated a publication from social scientists praising the “Mothers of IVF” and honoring patient 38 who endured 10 laparoscopies, achieving only an ectopic pregnancy for all her compliant efforts. Actually 11 women had 5 or more OPUs and deserve honorable mentions (10). In fact, the mothers of the two pregnancies that continued to surviving live births can be classified as “good prognosis” as their pregnancies resulted from a single laparoscopy. This implies that the other 280 women had endured a poor-prognosis (99.3%).

## Reducing the Poor-Prognosis Feature 1978–1982

Whilst IVF practitioners in modern day might dismiss the aforementioned history as “teething problems,” it is also relevant to point out that the ensuing 4 years were also difficult with only 9 pioneering groups worldwide (Table 1) reporting livebirths to July 1982 and a dozen by end 1982 (9). Of interest, almost all these units followed the Edwards dictum of pursuing natural cycles, but units in only 3 countries achieved livebirths from that protocol (4 units; Oldham UK; Royal Women's Hospital unit in Melbourne, Australia; the Clamart unit in Paris, France; and the unit at Sèvres, also in Paris, France. The Frydman unit at Clamart had developed a rapid plasma radio-immunoassay for LH as an advance over the HiGonavis test). All the others, as well as these 4 units eventually, abandoned Natural Cycle IVF for various forms of ovarian stimulation in IVF. In fact, the pioneering unit with the second successful live birth—the unit from from Kolkata (Calcutta), India with the birth of Kanuprija “Durga” Agarwal on 3 October 1978, applied ovarian stimulation (HMG) and other techniques which were at least 30 years in advance of then current IVF practice. These included the idea of embryo cryopreservation and subsequent frozen embryo transfer (FET) in a natural cycle. Of the other pioneer units shown in Table 1, eight used Clomid with HCG trigger and three applied HMG with HCG trigger. Only two units applied any luteal phase support, that being progesterone injections in the Norfolk, USA unit and the progestogen medroxyprogesterone acetate (MPA) in the Perth, Australia unit. Five of the pioneer centers reported twin pregnancies, three with livebirths in 1982 and two others with livebirths in early 1983. One of the latter was the first report of monozygotic twins from IVF.

**Table 1:** Lists the pioneer IVF centers which established livebirths from IVF, beginning with Louise Brown in July 1978 and documenting 9 successful centers to her 4th birthday (July 1982); thereafter another 3 centers to the end of 1982.

**Team**	**Country**	**City**	**Main members**	**First L/Birth**	**Ov Stim/Tr/LS^#^**
1	Britain	Oldham, Manchester, UK	Steptoe, Edwards, Purdy	Jul-78 Jan-79	Nil/nil/nil^$^
2	India	Kolkata/Calcutta, India	Mucherjee, Muckhergee, Battacharya	Oct-78	hMG/hCG/FET
3a	Australia	Melbourne, Australia	Wood, Johnston, Lopata	Jun-80	Nil/nil/nil
3b	Australia	Melbourne, Australia^*^	Wood, Leeton, Trounson	May-81 Jun-81	Clomid/hCG/nil 9 L/Bs, 10 infants, Twins
4	USA	Norfolk, Virginia, USA^*^	Jones, Seeger-Jones, Garcia, Acosta, Veek	Dec-81 Mar-83	hMG/hCG/P4 Inj Twins
5a	France	Clamart, Paris, France	Frydman, Testart, Lasalle, Papeirnik	Feb-82	Nil/nil/nil
5b	France	Sèvres, Paris, France	Cohen, Plachot, Mandelbaum	Jun-82	Nil/nil/nil
6	Britain	London, UK^*^	Craft, Yovich, Green, Shelton, Bernard	Apr-82	Clomid/hCG/nil Twins
7	Germany	Erlangen, Bavaria, Germany	Trotnow, Kniewald, Habermann	Apr-82	Clomid/hCG/nil
8	USA	Los Angeles, California, USA	Marrs, March, Mishell	Jun-82	Clomid/hCG/ P4 inj
9	Australia	Perth, Western Australia^*^	Yovich, Pusey, De Atta, Roberts, Reid, Grauaug	Jul-82 May-83	Clomid/hCG/MPA Twins (MZ)
10	Sweden	Stockholm, Sweden	Hamberger, Nilsson, Wikland, Enk	Sep-82	Clomid/hCG/nil
11	Israel	Sheba, Ramat Gan, Israel	Maschiach, Dor, Ben-Rafael	Sep-82	Clomid/hCG/P4 inj
12	Austria	Vienna, Austria^*^	Feichtinger, Kemeter, Szalay	Oct-82 Nov-82	Clomid/hCG/nil Twins

*References for the data in this table can be found in Yovich and Craft (9). These first IVF livebirths were “hard-won” with Team 3b generating the best results; from 115 initiated cycles 14 pregnancies (12.2%) and 9 deliveries ensued (7.8% live birth rate)*.

# *Ov stim/Tr/LS, Ovarian stimulation/Trigger/Luteal support*.

$ *nil/nil/nil, no ovarian stimulation/no trigger injection/no luteal support. P4 Inj, progesterone injections; MZ, monozygotic*.

* *The first IVF twin live births—in order 1. Melbourne, Australia; 2. London, UK; 3. Vienna, Austria; 4. Norfolk, Virginia, USA; and 5. Perth, Australia*.

## Reducing the Problem of Poor-Prognosis 1983–2003

In the two decades from 1983, IVF outcomes improved, meaning that livebirths were being reported from all over the world. Although pregnancies tended to be sporadic for start-up units, the live birth rates per initiated cycle for established facilities were rarely better than 10%. That decade was notorious for publications reporting variously irregular numerator and denominator criteria to provide the “best look.” Many units reported on favorable segments of practice. Therefore, it was generally not possible to know accurately whether IVF methodology was improving or whether the rising number of IVF babies was simply the result of IVF units selecting the younger, easier cases suited to the early protocols and laboratory methods (11). In fact, a cynical view might be that many newer start-up units were overstimulating young women who had highly responsive ovaries to boost positive outcomes. This period saw numerous cases of ovarian hyperstimulation syndrome (OHSS) and rising rates of high-order multiple pregnancies; the case of “Octomum” being the most notorious with 14 babies (4 singletons, 1 twin, and 1 octuplet) in one woman arising from a single initiated IVF cycle with ET and subsequent FET procedures (12). However, a true technical advance in IVF during this period was the introduction of ICSI in the early nineties (13), resulting in a solution for most male-factor causes of infertility and the potential avoidance of unexplained complete failed-fertilization (14). This advance broadened the indications for IVF and enabled the successful management of even azoospermic males when applied in concert with Vasal flush, PESA, MESA, and micro-TESE procedures (15, 16).

During this decade there were other definable progressive advances to IVF methodology which had the effect of further expanding the spectrum of infertility case scenarios which were responsive. In particular the introduction of gonadotrophin releasing hormone analogs, initially agonists (GnRHa) from the mid-1980's and later antagonists (GnRHant) in the mid-2000's. These introductions created control over the ovulation process, reducing elevated LH levels and preventing premature LH surges. This also enabled optimization of the ovulation trigger whereby ovulation could be delayed, to be triggered once maturation [based on ovarian follicle dimensions on pelvic ultrasound and serum estradiol (E2) levels] had been reached. The trigger injection included the use of GnRHa to replace HCG in cases managed with GnRHant who had high follicle numbers, with consequent near complete avoidance of OHSS (17).

In response to the several problems of OHSS, multiple pregnancies (with the associated problem of pre-term deliveries) and the complaints of high failure rates these two decades saw the increasing regulation of IVF practices. This was mostly self-regulatory by guidelines advised through societies such as the American Society of Reproductive Medicine (ASRM), the European Society of Human Reproduction and Embryology (ESHRE), the Fertility Society of Australia (FSA), and similar societies in most countries. In some countries there was a perceived need to introduce legislation resulting in statutory controls, such as the Human Fertility Embryology Act (1990) in the UK and similar Acts in some states of Australia. However, this type of regulation is now perceived to be unnecessary (18). A strong emphasis is now placed on teaching and training leading to improved laboratory methods which included the use of commercially prepared refined culture media, adapted for specific purposes i.e., flushing media, fertilization media, cleavage stage media, and blastocyst culture media (19). Specific cryopreservation media have also been developed for the advanced vitrification technique introduced from 2007 (20).

## Evolving Concept of Poor-Prognosis From 2004 to Current

Over the aforementioned historical period, the concept of “poor-prognosis” was largely changed by the development of IVF methodologies to ensure a high-quality clinical service in the setting of “tight” laboratory processes and continuous data evaluation enabling IVF clinics to rate their performance and be rated by independent assessment. In Australia and New Zealand this is enacted by an annual accreditation process by the Reproductive Technology Accreditation Committee (RTAC) acting under the auspices of the FSA. This self-regulatory system is strengthened by the requirement of accreditation so that patients attending the IVF unit can be eligible to receive the substantial Medicare benefits provided by the National Governments of Australia and New Zealand.

The improvement in cryotechnology has led to the consideration of a new treatment concept i.e., the segmentation of IVF treatment, with embryo transfer performed in subsequent frozen embryo transfer (FET) cycles. In practical terms this may result in a “freeze-all” cycle which commits all embryos (best at the blastocyst stage) to cryopreservation by Vitrification, and which is best applied using the Cryotop method (20). Whilst the idea of routine segmentation for all is not yet considered to be the best approach, many clinics in the Australian setting are currently committing their best blastocysts to the freezer, transferring the second tier in the fresh cycle. This approach is gaining popularity, contingent upon the data outcomes reported in the Australian and New Zealand Assisted Reproduction Database (ANZARD) (7) which reveals a higher live birth rate from FET cycles than fresh ETs (Table 2). However, despite all the aforementioned progress, ANZARD reports that the results of IVF across the 91 Fertility Clinics operating during 2017 in Australia (*n* = 83) and New Zealand (*n* = 8) vary widely. The productivity rate (live births per cycle initiated; fresh and frozen autologous) ranged from 9.3 to 33.2% for data covering 98% of clinics; i.e., all those 88 of 91 clinics undertaking >50 OPU cycles (7). The report provides no analysis for the wide range and data concerning adjuvants is not collected at this stage.

**Table 2:** Live birth outcomes from all fresh and frozen embryos transferred in Australia and New Zealand 2017.

**Parameter**	**Autologous cycles with fresh transfer (all ages)**	**Autologous thaw cycles (all ages)**
Initiated cycles	47,545	29,808
Cycles with OPU	42,632	–
Freeze-all cycles	12,110	–
Embryo transfer cycles	24,095	28,770
Clinical pregnancies	7,529	10,379
Live deliveries	5,803	8,310
Live deliveries per initiated cycle^#^	12.2%	27.9%^*^
Live deliveries per initiated cycle (excluding freeze-all cycles)	16.4%	–
Live deliveries per embryo transfer cycle	24.1%	28.9%^*^
Live deliveries per clinical pregnancy	77.1%	80.1%^*^

*Data extracted from ANZARD report [(7); Table 9; fresh IVF ± ICSI cycles and Table 13; FET cycles]. Shows that the live birth rates per initiated cycle as well as per embryo transfer procedures, are significantly higher for frozen embryo transfers; 89.4% single embryo transfers; 82.0% blastocyst-stage transfers, 91.5% being cryopreserved by vitrification. The live deliveries (births) per initiated cycle may be compared to the outcomes reported in Table 1 over the years 1978–1982, where the best from the first 12 IVF Teams was 7.8%; not much lower than the fresh cycle outcome of 12.2 and 16.4% shown here. Within this ANZARD report, Figure 1 shows the live birth delivery per initiated fresh (excluding freeze-all) and thaw autologous and recipient cycle among 88 of the 91 fertility clinics (i.e., those performing >50 OPU cycles for the year) ranged from a low of 9.3% to a high of 33.2% across all ages. Information about clinics use of adjuvants and add-ons is not available in the report but we are aware that GH was used in 22% of OPU cycles in one of the IVF centers reporting live births >30% of initiated cycles across all ages*.

# *At least one live infant at delivery; singletons 96.8%*.

* *p < 0.0001 Chi-square with Yates correction*.

## Changing Clinical Profiles of Couples Undertaking IVF

Historically IVF was applied for mainly underlying female factors classified as tubal, endometriosis, and other pelvic disorders; but increasingly clinics have added ovulation disorders and male factor infertility; the former to avoid multiple pregnancies arising from ovarian stimulation and the latter because of the introduction of ICSI. Nowadays, ANZARD (7) shows that 10% of cases are designated combined male and female factors, but the highest infertility categorization is “unexplained,” being more than 50% of cases. This phenomenon has led to some critical articles in the literature implying that IVF is being applied to many cases with inadequate workup; cases which might result in spontaneous pregnancies if managed better. One approach which increases the chances of avoiding IVF by careful workup, close monitoring and applying an Assessment Cycle has been published recently (21). Such a comprehensive approach identifies cases which can benefit by attention to nutritional health factors, coital timing, by offering oral therapies for disordered ovulation, by tubal flushing with lipiodol, by intra-uterine insemination (IUI) for negative post-coital tests and by hormonal supports where indicated during the luteal phase and early pregnancy. However, the most important factor for conception is female age, hence the argument about non-IVF treatments may hinge on available opportunity, such being greatest for young women <35 years and least for women ≥40 years.

## Ovarian Stimulation Schedules Influencing Poor-Prognosis

Further criticism of the Bologna criteria for POR, concerns the definitions of a standard and maximal stimulation schedule which, in the ESHRE context, means 150–225 IU rFSH. A more advanced dosage algorithm enabling a wider dosage range, targeted to multiple patient characteristics has been proposed (22) and subsequently validated by a prospective study within the same IVF unit (5). Such a targeted algorithm optimizes oocyte recovery to 10 ± 2 oocytes across the range of AFC and AMH categories and can improve the chance of live births, even in older women, with the effect of reducing the proportion of women labeled as poor-prognosis. A further, recently introduced, novel algorithm described as the POSEIDON stratification of low prognosis patients was recently proposed (23) and data is already beginning to appear which tends to validate its utility (24).

However, a confounder in the historical story of ovarian stimulation for IVF is the idea of minimal stimulation regimens which emerged during the 1990's when cases of OHSS were relatively common and included some reports of mortality. The idea was strongly promoted by clinics in Japan which reported “favorable” pregnancy rates per embryo transfer, without experiencing OHSS over 20 years, but not really disclosing the full story. However, the reality has been finally revealed by the Ethics Committee of the Japan Society of Obstetrics and Gynecology (25). Chairman Professor Saito shows that Japan has actually experienced the lowest live birth rate per initiated fresh IVF cycle reported anywhere in the world, the rate being 4.13% of OPUs undertaken in 2015. Furthermore, the productivity rate combining FET cycles is extremely low as the number of women having supernumerary embryos frozen (after fresh ET) is a very low proportion of the total initiated cycles as the majority have 0–4 oocytes recovered. These results can be contrasted with the ANZARD report which shows that in 2017, the SET rate in Australia and New Zealand was 89.4% and multiple pregnancies were a low 3.6%, being twins only without a single high-order live birth. This ensured that 80.2% of IVF infants were full-term singletons with normal birthweight. These favorable trends were achieved without any reduction in the live delivery rates, which actually increased to 26.8% per embryo transfer procedure (a significant rise from 22.5% over the 5-year period from 2012). There was a marked increase in the proportion of FET livebirths rising to 54.1% from 41.9% over the 5-year period with FET livebirth rates being 28.9% compared with 24.1% per ET for the fresh cycles. The markedly better FET implantation rates are shown in Table 2. The proportion of women undertaking IVF in the three age categories was similar being 36.6% for under 35 years; 36.2% for those aged 35–39 years; and 27.2% for those aged 40 years and over. Livebirth outcomes per initiated autologous cycle for those respective age ranges were 18.6% fresh and 32.0% frozen; 12.0% fresh and 28.0% frozen; 3.9% fresh and 17.3% frozen. Clearly, FET cycles generate more live births, a finding which is most marked with advanced female age. Furthermore, those pregnancies arising from FET cycles have a significantly higher chance of progressing to livebirths (80.1 vs. 77.1%; *p* < 0.001).

## Adjuvants in IVF

Notwithstanding the aforementioned progress in IVF methodologies, at least 10 categorical areas can be identified for improvement (Table 3). Many interventions (adjuvants, adjustments, and add-ons) have been introduced into the basic IVF model with a view to improving the chance of achieving a live birth from each cycle initiated, as listed in the table. These have included adjustments to improve ovarian responsiveness and the ovulatory response (to trigger), adjuvants to improve oocyte maturation, adjuvants to improve both implantation and placentation thereby diminishing pregnancy losses as well as add-ons for early pregnancy supports. The focus of this presentation is that of one adjuvant, namely GH. Although several of the add-ons listed in Table 3 are widely used, none have reached universal acceptance from the perspective of Cochrane (26, 27) or NICE (28) and their use has drawn rather scathing criticism (29, 30) because of the additional costs for unproven benefit.

**Table 3:** Listing adjustments, adjuvants, and add-ons which have been variously used in IVF and reported in medical literature.

**Adjustments, adjuvants, and add-ons in IVF**
**i. Response to ovarian stimulation**
a. Adjusting the schedule for LDR, Flare, and Antagonist protocols b. Adjusting the FSH dosing regimen c. Adding LH or HMG into the regimen d. The consideration of Clomid, Tamoxifen, or Letrozole
**ii. Ovulation trigger**
a. HCG dosage b. GnRha trigger c. Double trigger
**iii. Gamete preparation**
a. Sperm preparation and pentoxifylline enhancement b. Oocyte culture from GV to M-II (IVM) c. Growth hormone for improved oocyte quality d. Calcium ionophore A23187 e. ICSI despite normal semen analysis
**iv. Embryo assistance**
a. Zona hatching (mechanical, acid Tyrode, pronase solution, or laser method) b. Embryo glue (hyaluronin with recombinant human albumin)
**v. Luteal support protocols**
a. HCG b. Progesterone (P4) and progestogens c. E2/P4 combination pessaries d. GnRha
**vi. Implantation enhancement**
a. Low-dose aspirin ± heparin (anti-thrombotic) b. Antibiotics c. Enoxaparin/heparin d. Oral steroids (ACA, ANA, and LA antibody suppression) e. Verapamil (uterine relaxant, calcium antagonist) f. Acupuncture (stress relief, possible uterine relaxant) g. Atosiban (oxytocin/vasopressin Via receptor antagonist) h. G-CSF and Filgrastim i. C0-enzyme Q10 j. Dopamine agonists k. Intralipid (enhancing mitochondrial function) l. Testosterone (for low-androgen female) m. DHEA (for low-androgen female) n. Melatonin (strong anti-oxidant hormone) o. Growth Hormone (multifarious and ubiquitous actions) p. Endometrial scratch procedure q. Platelet infusion to uterine cavity r. Endometrial stimulation (GCSF-granulocyte colony stimulating factor) s. Paternal Lymphocyte immunization t. IVIG (intravenous Immunoglobulin)
**vii. Embryo selection**
a. Culture to blastocyst b. Quality grading of blastocyst c. Chromosomal evaluation for aneuploidy screening
**viii. Embryo-Endometrial synchrony**
a. Clinical calculation (Days from LH surge or P4 rise/ P4 supplements) b. Endometrial assessment (Endometrial receptivity array; ERA)
**ix. Early pregnancy supports**
a. Progesterone and progestogens b. Uterine relaxants (ß_2_-agonist e.g., ritodrine, salbutamol) c. HCG (enhance corpus luteal function) d. Low-dose aspirin (anti-thrombotic)
**x. Technical options**
a. Single lumen vs. flushing needle for follicle aspiration b. Ultrasound control for embryo transfers c. Myomectomy and pelvic surgeries pre-embryo transfers d. Tubal vs. uterine embryo transfers

*These adjuvants are categorized according to the putative area of action (noting HCG and Growth Hormone may apply in two areas—GH improving oocyte quality and possibly enhancing embryo implantation). Cochrane reviews have assessed all of these adjuvants and interventions, finding only aspirin and steroids demonstrating promising, potentially beneficial outcomes; but none yet proven to the highest level of EBM standards (26). Apart from one study including an “aspirin arm” and a second with a “DHEA” arm, none of the reported GH studies have considered these numerous potential confounders. G-CSF, granulocyte colony stimulating factor (filgrastim; G-CSF analog); GV, germinal vesicle; M-II, metaphase II; IVM, in vitro maturation; ACA, anti-cardiolipid antibodies; ANA, anti-nuclear antibodies; LA, lupus antibodies; LDR; long down regulation; FSH, LH, GnRHa in text*.

The historical preamble in this article was provided to show a poor-prognosis group has been evident in IVF from the beginning and that most of the useful developments for IVF to current day have been dependent on improving and tightening the protocols introduced in the early years. These can be summarized as focussing on the ovarian stimulation schedule and the trigger with a view to generating around 10 oocytes per IVF cycle thereby minimizing any risks to the woman. The translation of those 10 oocytes in best units is currently 1 good quality blastocyst-stage embryo transferred in the fresh cycle and an average of 2 blastocysts reaching sufficiently high grading to be vitrified for future FET attempts. This 30% oocyte utilization rate reflects current limited knowledge concerning oocyte maturation and controlling the age-dependent rate of chromosomal segregation errors occurring at the metaphase 1 stage (MI) which leads to aneuploidies in the embryo. Furthermore, the optimal luteal phase support has yet to be agreed upon (21) and ideas concerning early pregnancy management as well as the avoidance of pre-term delivery are only now appearing in the literature (31). The idea of evaluating a GH adjuvant trial must take these aspects into consideration to identify potential confounders.

## PIVET Experience With GH as an Adjuvant in IVF

Encouraged by the study on women aged >40 years which showed significant improvement of live birth rates by ovarian co-stimulation with GH in IVF (32), a GH adjuvant study was conducted at PIVET and the 5-year project was reported in 2010 (4). It was not an RCT but was designed as a prospective sequential crossover whereby patients identified as poor-prognosis were offered the option of using, or not using, GH in the forthcoming IVF cycle. (Some elected to use the expensive hormone in the immediate IVF cycle, others deferred depending on the outcome of a further non-GH cycle). Two protocols were explored using 10 IU ampoules given by injection in one of 2 protocols; Days 21 of previous cycle followed by Days 2, 6, 8, 10, and 12 of the IVF treatment cycle (60 IU over ~20 days); or Days 7, 14 and 21 of the previous cycle followed by a final injection on Day 2 of the IVF cycle (40 IU over ~35 days). Of 2,174 autologous IVF cycles during the period, 488 (22%) were classified poor-prognosis from previous experience providing 232 cycles started (initiated) with GH adjuvant and 256 without. The productivity rate was significantly higher among those poor-prognosis women given GH (43 vs. 11 live births; *p* < 0.001). However, the women classified as good prognosis had a productivity rate of 45.4% being well ahead of the poor-prognosis categories including those given GH 18.5%; *p* < 0.0001. From this study we understood that GH was safe for both mothers and offspring including the higher dosage regimen which encouraged its continued use. The benefits appeared similar from the two GH regimens described.

A second study from our PIVET facility was reported in 2017 (6) and again demonstrated an improvement in the quality of oocytes retrieved from 1,488 women categorized as poor-prognosis in IVF. The women were given dosages of 1.0 or 1.5 IU daily in the 6-week lead-up to OPU. This retrospective observational study showed a significantly higher oocyte utilization rate and embryo utilization rate among those women receiving GH compared to a computer-matched group of poor-prognosis cases who did not receive GH. This means a significantly higher number of oocytes become embryos which were either transferred as fresh ETs or cryopreserved for subsequent FET cycles. Among the case-matched women classified as poor-prognosis having fresh ETs the clinical pregnancy rate was 2.2-fold higher for Day-3 embryos and 7.6-fold higher for blastocyst transfers in those who had the GH adjuvant. This translated into an over-all improvement in live births of 6.2-fold for fresh ETs from the use of GH adjuvant (95% CI 2.8–13.4, *p* < 0.001). Of interest during this study a group of women classified as poor-prognosis chose the less-expensive oral Dehydroepiandrosterone (DHEA) option and others had combined DHEA with GH. In a separate report we failed to show any benefit from DHEA supplementation alone, neither any potentiating benefit or modification of the effect of GH treatment (33). Extending from this study, we subsequently reported on the outcome of treatments comparing the pregnancy outcomes of those cryopreserved embryos generated under GH influence with those arising without GH influence (34). Where FET cycles were carefully matched for age and poor-prognosis category, along with 6 other variables including embryo grading, AFC, AMH, BMI, and mid-luteal P4 levels, and analyzed by binary logistic regression, the live birth outcome was found to have improved significantly, being 2.7-fold higher (OR 2.71; *p* = 0.02) implying that GH had an influence beyond improving oocyte competence, extending to an embryo quality factor represented by enhanced competence to generate a live birth. Such embryos appeared morphologically similar to non-GH generated but outcomes were significantly better. We have suggested that such GH-generated embryos have an improved placentation capacity. Future proposed studies should also explore whether those embryos have any reduction in aneuploidy rates. However, the idea of GH being able to “fix” the problem of aneuploidy completely lacks scientific evidence.

## Global Studies With GH as an Adjuvant in Assisted Reproduction

The PIVET experience has been placed in context with 42 reported GH adjuvant studies from the year 2000 and summarized in Table 4 (4, 6, 32, 34–72). The first report in the table is from Nancy, France describing a single case with pan-hypopituitarism treated in a cross-over study (X-over refers to case studies where the outcomes were assessed on the same woman/women being treated with GH after cycles without GH). The woman was given GH 1 IU daily beginning 12 weeks prior to HMG stimulation and continuing through both the follicular stimulation phase and the luteal phase. GH was ceased following the detection of pregnancy 14 days after the HCG trigger injection (10,000 IU) with serum B-HCG of 462 IU/ml. A healthy male infant was delivered at 39 weeks with birthweight 3,780 g. This single case X-over report (35) should be compared with other cases of hypopituitarism such as the 4 cases from Rome, Italy (42) who had defined AGHD secondary to conditions such as brain trauma, empty Sella Turcica and Rathke's cyst. Normal fertility was established in these previously infertile women following “standard endocrine treatment” being weekly injections with GH averaging 0.5–1.0 IU daily given over a 3–6 month period, ceasing when pregnancy was diagnosed. In each of the 4 cases pregnancy outcomes were perfectly normal from all respects and the women successfully breast-fed their children. Two further single case X-over studies have also been reported, one from Brussels, Belgium (56) where a woman who had hypophysectomy and many IVF failures, conceived immediately from IVF following GH replacement therapy (~1 IU daily over 6 weeks). She delivered healthy twins at the 38th week of gestation. Of interest it was noted that her endometrial thickness had improved markedly under GH replacement therapy. The other similar single-case was recently reported from Bucharest, Romania (71). A woman with AGHD who had repeated failures from IVF, was given GH daily for 3 months in the lead-up to a further IVF treatment, this time resulting in better quality oocytes and a live birth.

**Table 4:** Forty-two studies published in English from year 2000 utilizing growth hormone in IVF programs.

**References *Spec feature***	**Type of study**	**GH v Con'l**	**GH dose**	**GH dur'n**	**Oocyte utilis'n**	**Embryo utilis'n**	**Pregnancy**	**Live births**
Salle et al. (35) *Hypopituitarism*	X-over	1 v 1	1 IU d	3 m	↑	↑	↑	↑
Sugaya et al. (36) *PORs*	X-over	9 v 9	4 IU d	4 w	↑	↑	ns	ns
Harper et al. (37) *Cochrane*	Syst Rev + M-analysis	154 v 150	4–8 IU daily	1–2 w	↑	↑	↑ OR 1.8	↑ OR 4.4^*^
Tesarik et al. (32) *women >40 y*	RCT	50 v 50	8 IU d	1 w	ns	ns	↑*P* < 0.05	↑*P* < 0.05
Menezo et al. (38) *GH in vitro*	Single case GV oocytes	GH to IVM	1.6 IU	stat	13/14 to MII	9/13 2PNs	1 B/C to FET	Healthy baby
Kyrou et al. (39)	Syst Rev + M-analysis	42 v 40	12–28 IU alt d	2–3 w	↑	↑	↑ OR nr	↑ OR 5.2
Guan et al. (40) *GH + aspirin*	RCT	20 v 20	nr	nr	↑	↑	ns	ns
Kucuk et al. (41)	RCT	31 v 30	12 d	3 w	↑	↑	ns	ns
Giampietro et al. (42) *GHD cases*	Observ	4 cases	3–6 IU w	6–12 m	Spont	Spont	Spont X4	Spont X4
Hazout et al. (43) *PORs*	Observ	245 v 2780	8 IU d	2 w	↑	↑	OR 1.5 *P* < 0.01	nr
Kolibianakis et al. (44)	Syst Rev + M-analysis	83 v 80	4–24 IU alt d	1–3 w	↑	↑	↑ OR 2.8	↑ OR 3.2
Yovich and Stanger (4) *Poor prognosis*	Prospec X-over	221 v 241	3–3.5 IU d	3 w or6 w	ns	ns	↑*P* < 0.001	↑*P* < 0.001
Duffy et al. (45) Cochrane	Syst Rev + M-analysis	148 v 131	4-8 IU d 24 alt d	1-3 w	↑	↑	↑ OR 3.3	↑ OR 5.4
Eftekhar et al. (46)	RCT	40 v 42	4 IU d	2 w	↑	↑	ns	ns
Haydardedeoglu et al. (47)	Retrospec	37 v 44	1.5–3 IU d	3 w	ns	ns	↑*p* < 0.001	↑*p* < 0.002
Hu et al. (48) PORs	Retrospec	102 v 287	4 IU	2 w	↑	↑	ns	ns
Lattes et al. (49) *PORs*	X-over	64 v 64	0.5 IU d	2 w	↑	↑	ns	ns
Dunne et al. (50) *Luteal phase GH*	Retrospec	14 v 28	10 IU d	2 w Pre IVF	ns	ns	ns	ns
Yu et al. (51)	Syst Rev + M-analysis	613 v 3,175	2–9 IU d average	1–3 w	↑	↑	ns	ns
Bayoumi et al. (52) *Microflare stiml'n*	RCT	72 v 73	8 IU d	2 w	↑	↑	nr	nr
Bassiouny et al. (53)	RCT	68 v 73	8 IU d	1 w	↑	↑	ns	ns
Wang et al. (54) *FET cycles HRT*	RCT (not strict)	77 v 77 v 76	4 IU d	2 w	↑ endometrial thickness		↑*P* < 0.03	↑*P* < 0.03
Du et al. (55) *NORs*	Retrospec	556 v 558	4.5 d	1 w	↑	↑	↑ OR 3.2	nr
Drakopolous et al. (56) *Hypopituitarism*	X-over	1 × 1	1 IU d	6 w	oocytes ↑ end thickness		↑ Twin preg	↑ 38 w
Ob'edkova et al. (57) *PORs*	Prospec observ	25 v 25	4 IU d	2 w	↑	↑	↑ OR 9.1	nr
Ho et al. (58) *3 arms: Age ≥40 y, RIF, PORs*	Retrospec Matched controls	98/36 118/118 33/33	3 IU d 2 IU d 2 IU d	2 w 2 w 2 w	Ns ↑ ↑	Ns ↑ ↑	ns *p* < 0.01 ↑*p* < 0.01	nr nr nr
Keane et al. (6) *poor prognosis*	Retrospec	161 v 239	1–1.5 IU/d	6 w	↑	↑	↑ RR 3.4	↑RR 6.2
Li et al. (59)	Syst Rev + M-analysis	320 v 343	1–12 IU d	1–3 w	↑	↑	↑ RR 1.8	↑RR 1.9
Altmae et al. (60) *RIF donor oocyte*	RCT	35, 35 v 35	nr	2 w	↑ endometrial thickness		↑ OR 6.9	↑ OR 6.4
Hart et al. (61)	M-analysis	351 v 352	M-analysis	1-2 w	↑ OR 1.9	↑ OR 1.5	↑ OR 1.5	ns
Cai et al. (62) *PORs*	Retrospec X-over	41/380 v 41/380	2 IU	6 w	↑	↑	ns	↑*p* < 0.003
Dakhly et al. (63) *PORs*	RCT	120 v 120	8 IU d	3 w	↑	↑	ns	ns
Chen et al. (64) *RIF cases*	Observ	22 v 20	2 IU d	2 w	↑	↑	↑*p* < 0.05	↑*p* < 0.05
Chu et al. (65) *Mild stiml'n*	Retrospec	61 v 71	2 IU d	2 w	↑	↑	ns	ns
Choe et al. (66) *Sust. Release GH*	RCT	64 v 63	S-R GH ~3 IU d	4 w	↑	↑	ns	ns
Regan et al. (67) *GC study*	Retrospec	13 v 10	2.5–3 IU d	3 w	↑	↑	↑	ns
Safdarian et al. (68) *PORs*	Retrospec	34, 32 v 26	0.5 IU −2.5 d	5d & 20 d	↑	↑	↑	ns
Cui et al. (69) *Thin endometrium*	RCT (?) FET cycles	40 v 53	?	2 w	↑ endometrial thickness		↑	↑
Norman et al. (70) *- recruited 130 of intended 390*	Double blind RCT	65 v 65	12 IU d	2 w	ns	ns	ns	ns
Keane et al. (34) *FETs*	Retrospec	109 v201	1–3 IU d	3–6 w	↑	↑	↑ OR 1.8	↑ OR 2.7
Albu et al. (71) *AGHD case*	X-over	1 v 1	1 IU d	3 m	↑	↑	↑	↑
Liu et al. (72) *NORs*	Retrospec Matched	781 v 781	2 IU d 4 IU d	2 w6 w	ns	ns	↑	nr

* *Harper et al. (37); 6 studies, OR highest when Howles 1999 GHRF study removed (OR 2.4–4.4). RCT, randomized controlled study; ns, not significant; nr, not reported; spont, spontaneous; Prospec, prospective; retrospec, retrospective; utilis'n, utilization; cont, control; trig, trigger; Observ, observational study; d, day; w, week; m, month; y, year; GC, granulosa cell; FET, frozen embryo transfer; RIF, recurrent implantation failure; NOR, normal ovarian responder*.

Seven of the reports in Table 4 document the results of Systematic Reviews (Syst Rev) and Meta-analyses. The Cochrane report of 2003 (37) documented the outcomes from 9 studies undertaken prior to the year 2000 (i.e., studies pre-Table 4). Six of those studies were undertaken on women classified with POR and three had unspecified classification for poor- prognosis. The first analysis showed no significant improvements but when trials using GH as the only adjuvant, particularly excluding a study combining growth hormone releasing factor (GRF), were separately analyzed, there was a significant increase in livebirths from 3 RCTs (OR 4.37). Five of the further Systematic Reviews with Meta-analyses (39, 44, 45, 51, 61) all revealed improved oocyte and embryo utilization and 3 reported significantly higher live births (ORs 3.2, 5.4, and RR 1.9). However, two separate M-analyses (51, 61) showed no increase in live births although oocyte and embryo utilization were significantly improved (OR 0.8 and 1.5; and OR 1.9 and 1.5, respectively). The report from Anhui, China (51) comprised 20 GH studies including one pre-2000 and 12 studies from various locations in China, some reports not fully detailed in English. Others are confused by alternative spelling of lead author e.g., Guan vs. Qun (40) and selection of family name for lead author e.g., Xue-Li Li vs. Li X-L (57).

Excluding the 6 X-over reports and the single-case IVM study (38), there were 28 controlled studies, 19 of which were categorized as retrospective or prospective / observational and 12 were RCTs, with 10 describing strictly random allocations. Of the non-RCTs, two-thirds reported significantly elevated oocyte and embryo utilization rates which translated to increased pregnancy rates in most, but live-birth rates were significantly improved in only 50% of the studies (9 but 2 others reported increased pregnancy rates but did not record births). Three of the 9 non-RCT studies showing improved live births emanated from PIVET (4, 6, 34) and the potential reasons for the varied outcomes among the global studies will be discussed (below). From the 12 RCTs, oocyte and embryo utilization were significantly elevated in all but one study, sometimes with an increase in pregnancy rates but only 5 of the 12 studies reported any significant elevation in live birth rates (32, 45, 54, 60, 69), three of which were focussed on endometrial enhancement (54, 60, 69).

The one RCT which failed to show any benefits from the GH adjunct (70) deserves specific scrutiny, as it was a registered study with which PIVET participated. In 2010, a multicenter, double-blind placebo-controlled trial was established in Australia and New Zealand with 10 participating IVF centers. Women had to be younger than 41 years, with demonstrated POR from previous IVF and have body mass index not >32 kg/m^2^ and baseline FSH level no higher than 15 IU/L. The trial was registered as the LIGHT (Live birth, *in-vitro* fertilization & GH Treatment) study and intended to recruit 390 IVF couples to provide 195 participants in each arm. This number would have provided statistical power at the 5% significance level if the live birth rate improved from a base level of 10% to an enhanced level of 20%. Both the GH hormone pen and the placebo pen were identical in appearance and both the patient as well as her medical attendants were blinded to the active vs. inactive injection. The GH dosage was 12 IU to be given concomitant with the gonadotropin, meaning approximately 12 days (actual range 11–13 days) of injections; ~144 IU total GH. However, the LIGHT study closed after 8 years effort having recruited only 130 couples into actual treatment, being only a third of the number required. As we have earlier indicated, where patients are paying for treatment, they are reluctant to risk being allocated to the placebo arm, even though the GH hormone was provided without charge. They surmised that there would be a loss of monies and opportunity, particularly if they were aged in their late thirties. Nonetheless, the data from the 130 women has been analyzed and published (69) with the report showing no improvement in oocyte number or utilization; no improvement in embryo number or utilization and no difference in either pregnancy rate or live birth rate. The only difference demonstrated between the two arms of the trial was the finding that GH patients reached oocyte retrieval faster than non-GH patients, similar to that shown in other earlier studies (44, 60). This may indicate that GH has an effect on folliculogenesis; however, this possibility was not supported by differences in embryo quality. Nonetheless, the links between GH and folliculogenesis, oocyte quality and responsiveness to gonadotrophins is still unclear from this study, being underpowered, focussed only on POR cases. That study does not yet report outcomes from cryopreserved embryos, although we would not hold high hopes for these as they were all cryopreserved in slow-freeze protocols prior to the introduction of vitrification to Australian IVF facilities. The authors acknowledge these points but also conclude with a negative comment*: “In conclusion, this study does not show increased efficiency of human GH as an adjunct to FSH treatment in subjects receiving IVF who have been previous poor responders.” They caution women against expenditure in this area, citing their own earlier Meta-analysis which showed no pregnancy or live birth benefit for POR cases* (61). Furthermore, the National Institute for Health and Care Excellence (Nice) has issued clinical guideline CG156 recommending “Do not use growth hormone or dehydroepiandrostenedione (DHEA) as adjuvant treatment in IVF protocols” (73). This recommendation is based on a dogmatic EBM attitude that an appropriately structured RCT has not demonstrated a benefit; however we would argue that such a study is not feasible in the current circumstances.

Our perspective is described in response to another recent study, that from the well-published group from Cairo, Egypt (63). Their registered RCT, albeit with borderline numbers (120 in each arm), trialed a 3-week course of GH 7.5 IU daily in POR cases. They showed a significant improvement in both oocyte utilization and embryo utilization, meaning more embryos were transferred or cryopreserved in the GH arm. However, these improvements did not translate into more pregnancies or more livebirths, either from fresh cycles or from the added FETs (cumulative live births; live birth productivity rate). In a letter to the same journal we pointed out several limitations which could have limited their outcomes (74).

## Optimizing IVF Outcomes; a Single-Center Viewpoint

We would summarize the current requirements to maximize the opportunity for embryos to implant and achieve higher LBRs, thereby reducing the poor-prognosis rate in IVF, requires adherence to the previously mentioned protocols (21), namely:

1. Apply PIVET FSH dosing Algorithms to optimize oocyte numbers (at 10 ± 2)
2. Apply SET protocol for all cycles, especially the fresh cycle.
3. Blastocyst culture preferred, with best quality embryos vitrified by Cryotop method
4. Strong luteal support in fresh cycle using P4 pessaries ± HCG with monitoring, enabling adjustment of dosages. Optimal early-luteal serum progesterone levels should range from 60 to 100 nmol/l (75) rising to between 150 and 250 nmol/l in the mid-luteal phase. Sometimes P4 injections will be required
5. FET cycles conducted under either natural cycles or HRT (for logistic benefits). Optimal P4 levels should be between 60 and 100 nmol/l in the mid-luteal phase (76)
6. FET with SET preferred with PIVET regimen: P4 Pessaries ± P4 injections
7. GH adjuvant therapy for IVF cases diagnosed with AGHD.

The last point (#7) may be considered controversial with strong skepticism about GH use expressed by several prominent clinicians in the field (70, 77, 78) excepting when used for women with hypopituitarism. Such can be due to a range of anatomical causes such as empty Sella Turcica, pituitary adenomas, Rathke's cyst/pouch, hypophysectomy, and other intracranial trauma as well as medical conditions such as Sheehan's syndrome. Such cases respond dramatically well to growth hormone replacement therapy 1 IU daily for 3–6 months as shown from the 6 X-over studies in Table 4. On the other hand, equally prominent IVF clinicians (79) are perplexed that GH is under-utilized given that “the most recent Meta-analysis (59) shows almost double live birth rates in those poor responders and/or couples with a reduced prognosis.”

## Concluding Viewpoint

Our concluding viewpoint is that GH is clearly indicated in those women with infertility where this can be shown to be due to AGHD. This condition is currently under-diagnosed, but can be determined by applying screening tests involving IGF-1 and its main binding protein IGFBP3 (8). Endocrinologists may utilize sophisticated challenge tests where the diagnosis is uncertain, but IVF specialists may apply a simpler screening where there is already clinical evidence such as advanced female age and repetitive failure to generate any blastocysts for vitrification (i.e., defined poor-prognosis). The Bologna screening of POR has several limitations with respect to the application of GH, many of which have been discussed earlier in this article. In particular POR may often represent a highly depleted ovarian reserve, and such is an impossible challenge for GH and the inclusion of such cases may well explain the variable outcomes of the GH trials indicated in Table 4. We would believe that GH would apply best to those women who do respond to high-dose gonadotrophin injections (generating 8–12 oocytes with FSH doses up to 450 IU), but who fail to generate sufficient blastocysts of suitable quality to enable at least one or two for cryopreservation, after the transfer of one embryo in the fresh cycle. Where a freeze-all option is contemplated, there should be at least 2 high-grade embryos cryopreserved, otherwise the case can be classified as poor-prognosis, warranting GH therapy. With respect to the dosage and duration of GH therapy, our experience over more than 12 years indicates that 1 IU daily is sufficient to produce a response (e.g., raising IGF-1 levels) but an optimum response with respect to oocyte quality probably requires 4–6 months to cover the full period of folliculogenesis from the earliest stage of primary follicle recruitment being at least 20 weeks prior to the ovulatory cycle, when paracrine controls over oocyte development are strongest (80). We acknowledge there may be both logistic and financial problems to such a prolonged treatment schedule, hence a compromise treatment proposal could be a six-week schedule, beginning Day 2 of the menstrual cycle preceding the IVF cycle. Perhaps an increased dosage of GH at 2 IU might be a rational consideration given that the pioneer studies in the 1980's showed a dose-related effect on both follicle growth and IGF-1 levels (8, 81). A further notion that GH may help to preserve the primary follicle pool is appealing but awaits specific research. Such an idea implies that older women (e.g., >35 years) who wish to preserve their fertility, might benefit from continuous long-term GH therapy. Whilst studies investigating this idea have never been reported, there are reports that women who had GHD as children, will have health and fertility benefits from continuing GH therapy after puberty, the current stage of cessation (8). We believe there is sufficient data currently showing that GH can have a beneficial effect in IVF programmes but further research is required to forecast which woman will be deemed poor-prognosis, how such may be prevented, which cases will benefit from GH, and what therapeutic regimen should be applied for optimal management.

## Author Contributions

JY heads this research team involving Clinical (JY and YY) and laboratory studies (SR and KK). Data analysis and manuscript preparation is shared by JY and KK.

### Conflict of Interest

The authors declare that the research was conducted in the absence of any commercial or financial relationships that could be construed as a potential conflict of interest.
